# Binding characteristics of chemosensory protein 11 from *Grapholita molesta* Busck (Lepidoptera: Tortricidae) to insecticides

**DOI:** 10.7717/peerj.21510

**Published:** 2026-07-20

**Authors:** He-Fen Nian, Guang-Wei Li, Shao-Qiu Ren, Kun Luo, Xiu-Lin Chen, Bo-Liao Li

**Affiliations:** Key Laboratory for Applied Ecology of Loess Plateau (Shaanxi Province), College of Life Sciences, Yan’an University, Yan’an, Shaanxi Province, China

**Keywords:** Chemosensory protein, Insecticide resistance, Fruit moth, Fluorescence competition assay, Molecular dynamic stimulations

## Abstract

Chemosensory proteins (CSPs) are small soluble proteins that recognize and bind to various small chemical molecules. Although numerous studies have shown that CSPs play an important role in olfactory communication among insects, recent research indicated that CSPs also contribute to insecticide resistance. The antennae-enriched GmolCSP11 from *Grapholita molesta* Busck exhibits high binding affinities for seven insecticide ligands, as verified by fluorescence competitive binding assays of recombinant protein. GmolCSP11 exhibited the strongest binding affinity for chlorpyrifos (*K_i_* = 1.01 ± 0.00 μM). Molecular docking and molecular dynamics simulation revealed that the stable formation of GmolCSP11-chlorpyrifos primarily relies on van der Waals interactions between amino acids, including Tyr25, Phe42, Leu64, Ile65, and chlorpyrifos. Site-directed mutagenesis analysis revealed that mutating any of the aforementioned four amino acids to alanine significantly decreased the binding affinity for chlorpyrifos. This study could contribute valuable insights into how moth CSPs recognize and sequester insecticides, potentially offering potential avenues for developing novel pest management strategies.

## Introduction

Insects live in complex and dynamic environments in nature, leading to the evolution of their olfactory system that enables the recognition of diverse chemical signals from the environment (Ebrahim et al., 2015). Proteins in the olfactory system of insects comprise chemosensory proteins (CSPs), odorant binding proteins (OBPs), odorant receptors (ORs), ionotropic receptors (IRs), sensory neuron membrane proteins (SNMPs), and odorant degrading enzymes (ODEs) (Leal, 2013; Renou & Anton, 2020; Duan et al., 2022). CSPs, together with OBPs, are small soluble binding proteins that are highly concentrated in the lymph of the chemosensillar and are believed to transport odor pheromone molecules from the antenna surface to the chemosensory neurons’ membrane (Pelosi et al., 2018). CSPs have a three-dimensional structure featuring six α-helices and four conserved cysteines that form two disulfide bonds (Lartigue et al., 2002; Pelosi et al., 2014). Initially, insect CSPs were considered to be olfactory fragment D (OS-D) or sensory appendage proteins (SAPs) due to their high expression in the chemosensory tissues (McKenna et al., 1994; Robertson et al., 1999). Tremendous CSP sequences have been identified with the widespread adoption of RNA-seq sequencing and genome sequencing techniques (Jing et al., 2020; Cui et al., 2022; He, Crabbe & Ren, 2023; Zhou et al., 2024).

Because insect CSPs are highly expressed in antennae, it is logical that functional research on CSPs mainly focuses on the recognition and reception of plant volatile odorant or pheromone molecules. Recent studies, however, reveal that CSPs function in broader roles than previously understood, including behavioral phase change (Guo et al., 2011), development (Maleszka et al., 2007), feeding behavior (Yi et al., 2017), avoidance of fungi pathogen (Zheng et al., 2023), and sequestering of insecticides or toxic xenobiotic molecules (Liu et al., 2016). For instance, the abilities of four antennae-expressed CSPs to bind insecticides demonstrated that SlituCSP18 is more sensitive to pesticide treatment and may play a key role in how the olfactory system and the pesticide interact (Lin, Mao & Zhang, 2018). PxylCSP1 contributes to indoxacarb resistance in *Plutella xylostella* (Li et al., 2023b). RNAi knockdown of *RpCSP4*, *RpCSP5*, *RpCSP6*, and *RpCSP10* greatly increased the mortality of *Rhopalosiphum padi* upon exposure to imidacloprid or beta-cypermethrin (Peng et al., 2021). Suppression of *RpCSP4* and *RpCSP5* transcription by RNA interference significantly heightened the susceptibility of *R. padi* to thiamethoxam (Gao et al., 2023a; Gao et al., 2023b). Overexpression of the *RpadCSP7* contributed to lambda-cyhalothrin resistance in *R. padi* (Gao et al., 2023a). In research on the CSPs of another aphid, *Aphis gossypii*, the results showed that the expression of *AgoCSP1*, *AgoCSP4*, and *AgoCSP5* was upregulated by cyantraniliprole treatment. Suppressing these genes by RNAi induced higher mortalities in *A. gossypi* (Xu et al., 2022). The *Drosophila* flies overexpressing *AgoCSP5* from *A. gossypi* demonstrated reduced susceptibility to omethoate, imidacloprid, and cypermethrin (Li et al., 2021). These findings collectively underscore the critical roles of CSPs in insecticide resistance, suggesting that manipulating CSP expression could have significant implications for pest control.

The oriental fruit moth, *Grapholita molesta* Busck (Lepidoptera: Tortricidae), poses a significant economic threat to stone and pome fruit production worldwide, with a wide distribution in temperate and subtropical regions of Asia, Europe, and Oceania (Myers, Hull & Krawczyk, 2006; Wei et al., 2015; Zhang et al., 2022). Its larvae inflict damage by feeding on young twigs and buds, as well as directly on the fruit pulp (Duarte et al., 2015). Currently, chemical pesticides are the primary method for controlling *G. molesta* (Kanga et al., 2003; Najar-Rodriguez, Bellutti & Dorn, 2013). This reliance on insecticides in orchards has driven the evolution of insecticide resistance in this fruit moth. Insecticide resistance mechanisms include penetration resistance (Balabanidou, Grigoraki & Vontas, 2018), target resistance (Casida & Durkin, 2013), metabolic resistance (Pavlidi, Vontas & Van Leeuwen, 2018; Walsh et al., 2022), and sequestration (Pavlidi, Vontas & Van Leeuwen, 2018). However, the sequestering mechanism, despite its significance, has received comparatively less attention than metabolic insecticide resistance in *G. molesta* (Han et al., 2022; Li et al., 2023a; Zhang et al., 2023).

Recent research has demonstrated that recombinantly expressed CSPs can selectively bind to hydrophobic insecticide molecules (Li et al., 2021; Li et al., 2023b; Gao et al., 2023b; Gao et al., 2024b). Li et al. (2015) have identified 17 CSP genes from RNA-seq data using *de novo* assembly (NCBI SRA accession: SRX604493). After the genome of *G. molesta* was published (Yang et al., 2025), we re-analyzed this data and identified an additional six CSP genes. As the larvae of *G. molesta* bore into fruits or twigs, which are not easily killed by contact insecticides, the adult stage should be a key period for pest control. Among all these CSP genes, GmolCSP11 was almost exclusively expressed in antennae and was sensitive to insecticide-induced stress. The current study aims to clarify the role of GmolCSP11 in insecticide tolerance. We used prokaryotic expression and fluorescence competitive binding assays to quantify the binding affinities of GmolCSP11 for different insecticide ligands. Moreover, we identified key binding sites of GmolCSP11 to seven insecticides through homologous modelling, molecular docking, molecular dynamics simulation, and site-directed mutagenesis, in combination with ligand-binding assays. These findings are expected to provide valuable insights into the contribution of CSPs to the enhancement of insecticide resistance in *G. molesta*.

## Materials & Methods

### Insect rearing and tissue collection

The larvae of *G. molesta* were collected in early June 2021 from plum orchards on the outskirts of Yan’an, China. Wandering larvae were transferred to moist sandy soil for pupation. The pupae were cultivated in the laboratory at 25 ± 1 °C, the relative humidity of 70 ± 5%, and a photoperiod of 15 L: 9 D. The emerged adult moths of both sexes were housed in plastic cups (600 mL) covered with perforated polyethene (PE) film with fine holes to ensure ventilation. The adults were fed with a cotton ball immersed in 5% honey water solution. The antennae from 200 males and 200 females were collected from 3-day-old virgin adults of both genders and immediately stored at −80 °C.

### RNA extraction, cDNA synthesis

Total RNA from each sample was isolated using AG RNAex Pro Reagent (Accurate Biotechnology Co., Ltd, Changsha, China), following the manufacturer’s instructions. The integrity and purity of the isolated RNA were determined using 1% agarose gel electrophoresis and a Nano-300 Micro-Spectrophotometer (Allsheng, Hangzhou, China). The first cDNA strand was synthesized from one µg of total RNA through an EasyScript One-Step gDNA Removal and cDNA Synthesis SuperMix Kit based on the instructions (TransGen Biotech, Beijing, China). The synthesized cDNA was either immediately used for PCR amplification or stored at −20 °C.

### Multisequence alignment

The amino acids of GmolCSP11 that were translated from GmolCSP11 (NCBI accession: KR003783.1) (Li et al., 2015) and CSPs from the other moth species were aligned using MUSCLE (Edgar, 2004). The amino acid sequences of CSP11 from *G. molesta* and other moths were compared using the ESPript3 online server (https://espript.ibcp.fr/ESPript/ESPript/) (Gouet, Robert & Courcelle, 2003) and the SignalP-6.0 server (http://www.cbs.dtu.dk/services/SignalP/) (Teufel et al., 2022) was used to search for conserved Cys residues and predict N-terminal signal peptides of GmolCSP11, respectively.

### Real-time quantitative PCR (RT-qPCR) and data analysis

RT-qPCR was conducted using a StepOnePlus™ Real-Time PCR System (ABI, Carlsbad, California, USA) to compare the relative expression of *GmolCSP11* in various tissues (antennae, head without antennae, thorax without legs and wings, abdomen, legs, and wings) of 3-day-old male and female *G. molesta* adults. The reactions were performed in a 96-well plate. *β-actin* from *G. molesta* served as the reference gene (Li et al., 2015). Primer specificity and amplification efficiency (E) were assessed through a five-fold serial dilution of mixed male and female antenna cDNA. The primers for RT-qPCR were designed using Primer-BLAST (https://www.ncbi.nlm.nih.gov/tools/primer-blast/), and were listed in Table S1 (Ye et al., 2012).

Each 20 μL reaction consisted of 200 ng of cDNA template, 0.4 μL of ROX Reference Dye I, 0.8 μL of each forward and reverse primers (10 mM), 10 μL of qPCR SuperMix (2 ×) (TransGen Biotech, Beijing, China), and 7.0 μL nuclease-free water. Three biological replicates were conducted for each tissue sample, with each replicate undergoing three technical replicates. The qRT-PCR reaction program included a pre-denaturation at 94 °C for 30 s, followed by 40 cycles of amplification (94 °C for 5 s, 55 °C for 15 s, and 72 °C for 10 s), and a final extension at 95 °C for 15 s. Melting curve analysis was performed post-reaction to confirm the specificity of amplification. The qRT-PCR data analyses were carried out using the 2^−^^△△^C_T_ method (Schmittgen & Livak, 2008). One-way analysis of variance (ANOVA) was used to compare the relative expression across different tissues, and an independent *t*-test (α = 0.05) was used to determine sex-specific expression differences using R (v. 4.3.2). Results are exhibited as mean ± *SD*.

### Expression and purification of recombinant GmolCSP11 protein

Specific primers incorporating restriction enzyme sites were designed to amplify the DNA fragments encoding GmolCSP11 without signal peptides. Polymerase chain reaction (PCR)-amplified GmolCSP11 was verified by using 1.0% agarose gel and extracted using a Gel Extraction Kit (BioFlux, Hangzhou, China). The purified products were ligated into the pMD™19-T/(TA/Blunt-Zero) cloning vector (TaKaRa, Shiga, Japan), and transformed into DH5α competent cells (TransGen Biotechnologies, Beijing, China). Positive clones were selected and then screened by PCR. Plasmids containing the GmolCSP11 (pMD™19-T-GmolCSP11) were extracted using the GeneJET Plasmid Miniprep Kit (Thermo Scientific, Vilnius, Lithuania). After verification, pMD™19 T-GmolCSP11 plasmids were subjected to double-digestion with restriction endonucleases Hind III and NcoI for 4 h at 37 °C. Recombinant GmolCSP11 fragments were ligated into the expression vector pET28a (+) using T4 DNA ligase (ThermoFisher Scientific, Vilnius, Lithuania) overnight at 4 °C.

The ligated products were transformed into *Escherichia coli* competent cells, and positive clones were selected based on colony PCR. The pET28a(+)-GmolCSP11 plasmids were extracted and transferred into BL21 (DE3) competent cells (TransGen Biotech, Beijing, China). The cells were cultured in LB liquid medium with kanamycin (50 μg/ml) for 15 h under oscillatory conditions. When the culture OD_600_ reached 0.6 to 0.8, the bacterial cells were induced to express by adding isopropyl-*β*-D-thiogalactoside (IPTG) overnight at 28 °C and 220 rpm to a final concentration of 0.5 mM. The GmolCSP11 was validated using SDS-PAGE and identified as inclusion bodies. The bacterial cells were harvested (10 min at 8,000 g, 4 ^∘^C) after incubation for an additional 5 h at 37 ^∘^C with shaking at 220 rpm. The resuspended cellular pellets were first lysed with lysozyme (0.4 mg/mL), followed by sonication in lysis buffer (one mM phenylmethanesulfonyl fluoride, 250 mM NaCl, and 20 mM Tris-HCl pH 7.4), and subsequently centrifuged (12,000 g, 30 min, 4 ^∘^C). The denaturation and renaturation processes were conducted as follows: (1) The inclusion bodies were dissolved in solution I (20 mM Tris-HCl, pH 7.4, and 0.2% Triton X-100), centrifuged at 12,000 g for 20 min, and the supernatant was discarded. (2) The pellet was resuspended in five mL of 8 M urea solution, to which five mL of solution II (200 mM Tris-HCl, pH 8.0, and 10 mM dithiothreitol (DTT)) were added, and the mixture was maintained at room temperature. (3) 1.6 mL of solution III (0.5 M NaOH and 5 mM cystine) was added and incubated for 10 min. (4) A ten-fold volume of solution IV (100 mM Tris-HCl, pH 8.0, and 5 mM cystine) was added, and the mixture was incubated at 22 °C for 24 h at a rotational speed of 70 rpm. Following this, the mixture was centrifuged at 6,000 g for 30 min to finally obtain recombinant GmolCSP11. After proteins were purified by standard Ni-nitrilotriacetic acid (Ni-NTA) resin (Genscript Biology Company, Nanjing, China) to obtain a soluble protein of about 13 kDa size, the protein was recovered in 20 mM Tris-HCl (pH 7.4) by dialysis and used for *in vitro* fluorescence competitive binding assays. The purity and concentration of GmolCSP11 were verified by SDS-PAGE, and the protein concentration was measured using the BCA protein assay kit (Vazyme, Nanjing, China).

### Fluorescent competitive binding assay

The fluorescent competitive binding assays were conducted using an F-2700 spectrophotofluorometer (Hitachi, Tokyo, Japan) to evaluate the binding affinities of GmolCSP11 to seven insecticides, namely chlorpyrifos, indoxacarb, thiodicarb, beta-cypermethrin, lambda-cyhalothrin, avermectin, and spinetoram. The purities and sources of these seven insecticides are listed in Table S2. At the beginning, the fluorescence probe N-Phenyl-1-naphthylamine (1-NPN) and the tested ligands were dissolved in chromatographic methanol to create 100 mM stock solution, which was subsequently diluted to one mM for assays. GmolCSP11 protein was diluted to two μM in 20 mM Tris-HCl buffer (pH 7.4), and two mL of this solution was titrated with 1-NPN to a final concentration of 18 μM. The binding affinity was determined by measuring fluorescence intensity (excitation at 337 nm, emission scanned from 370–550 nm). As the concentration of 1-NPN was elevated, a corresponding enhancement in intensity was observed, which eventually plateaued. Upon stabilization, the data were recorded and subsequently utilized to ascertain the binding dissociation constant *K*_1−*NPN*_ (*K*_*d*_) of 1-NPN to GmolCSP11. Then, two ml protein solution containing two μM rGmolCSP11 was titrated with one μM of each ligand to a final concentration of 16 µM. The fluorescence intensity was measured and recorded after a 2-minute reaction. The inhibition constant (*K*_*i*_) for competitive binding of each ligand to GmolCSP11 was calculated using the formula *K*_*i*_ = [*IC*_50_]/(1+[*1*−*NPN*]/*K*_1−*NPN*_), where *IC*_50_ is the ligand concentration corresponding to 50% displacement of 1-NPN. Each assay was replicated three times biologically. The *K*_*d*_ and *K*_*i*_ are presented as mean ± *SD*.

### Homology modelling and molecular docking

Homology modelling of the three-dimensional (3D) structure of GmolCSP11 was performed using Swiss Model (https://swissmodel.expasy.org/) (Waterhouse et al., 2018), employing the 3D structure of CSP from the light brown apple moth, *Epiphyas postvittana* Walker (Lepidoptera: Tortricidae), (alphafold DB model: A0A0K8TW01.1.A) as a template. The predicted structure of GmolCSP11 was verified by ERRAT and Ramachandran plot using SAVES v6.1 server (https://saves.mbi.ucla.edu/). The 3D structure files of insecticide ligands for GmolCSP11 were downloaded from Pubchem database (https://pubchem.ncbi.nlm.nih.gov/) (Kim et al., 2023). Additionally, molecular docking was applied on the CB-Dock 2 server with the default settings (Liu et al., 2022). The structures of GmolCSP11-Ligands were visualized and edited by PyMOL-open-source (v2.5.0) (https://github.com/schrodinger/pymol-open-source).

### Molecular dynamics (MD) simulation and computational alanine scanning (CAS)

The MD simulations of GmolCSP11-ligand complex were carried out for 400 ns using GROMACS (v. 2023.3) (Abraham et al., 2015) with the AMBER99SB protein forcefield (Showalter & Brüschweiler, 2007). The simulation started with solvating the complex to neutralize the system. Energy minimization was achieved using the steepest descent algorithm with a step size of 0.01 nm. System equilibration was performed under *NVT* and *NPT* ensembles for 2 ns. After that, 400 ns production molecular dynamics simulations were performed twice. Analyses of root-mean-square deviation (RMSD), root-mean-square fluctuation (RMSF), radius of gyration (Rg), distance between centroid of ligand binding cavity and centroid of ligand were executed using GROMACS (v. 2023.3) built-in tools (Abraham et al., 2015), and visualized using the ggplot2 package (v. 3.4.3) (Wickham, 2016) in R (v. 4.3.2). Then, the binding energy for protein and ligands was calculated using the gmx_Molecular Mechanic and Poisson–Boltzmann Surface Area (gmx-MMPBSA) tool (Valdés-Tresanco et al., 2021). With the AlaScan module of the FoldX program, we performed mutation simulations on candidate amino acid residues of GmolCSP11 to assess their dominant energetic contributions (Delgado et al., 2019).

### Site-directed mutant and expression of mutants

Four site-specific mutations of GmolCSP11 (Y25A, F42A, L64A, and I65A) were generated based on the results of homology-based modelling and molecular docking analysis. These mutations were introduced into the pET-28a (+)-GmolCSP11 plasmid using the Fast Mutagenesis System (TransGen Biotech, Beijing, China). Site-directed mutagenesis primers were designed according to the specific mutation requirements (Table S1). The expression and purification processes were carried out identically to the methods described previously for the wild-type GmolCSP11.

## Results

### Sequence analysis of GmolCSP11

A full-length cDNA of *GmolCSP11* was cloned from the antennal transcriptome of the oriental fruit moth and verified by Sanger sequencing. *GmolCSP11* has an open reading frame (ORF) of 372 bp (NCBI accession: KR003783.1), encoding 123 amino acid residues with a signal peptide of 17 amino acids at the N-terminal (NCBI accession: ALC79597.1). The molecular weight (*MW*) and isoelectric point (*pI*) of the GmolCSP11 protein without signal peptide are 12.44 kDa and 6.71, respectively.

### Tissue expression profiles of GmolCSP11

**Figure 1:**
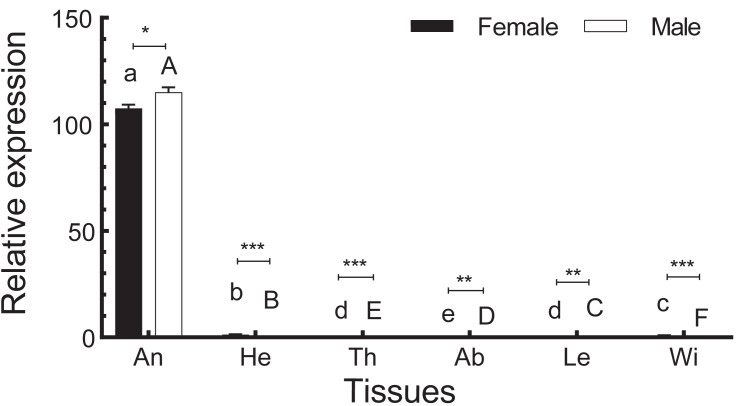
Relative expression levels of *GmolCSP11* in different tissues of 3-day-old adult *Grapholita molesta*. An, Antennae; He, Heads; Th, Thoraxes; Ab, Abdomens; Le, Legs; Wi, Wings. Different lowercase and capital letters above each histogram denote significant differences in expression levels between tissues in female and male adults, respectively (one-way ANOVA combined with Tukey’s multiple comparison, *α* < 0.05). Asterisks above the histogram indicate significant gender-based differences in the expression levels within the same tissue (*P < 0.05; **P < 0.01; ***P < 0.001; *t*-test).

The expression of *GmolCSP11* in various tissues of male and female adults was analyzed using RT-qPCR. The results indicated significant differences among tissues (Female: *F* = 9246, *df* = 5, *P* < 2 ×10^−16^; Male: *F* = 9744, *df* = 5, *P* < 2 ×10^−16^) (Fig. 1), with the antennae showing notably higher expression levels compared to other tissues. In addition, moderate expression levels were observed in the heads and wings of females. A comparison of *GmolCSP11* expression across the same tissues between sexes revealed significantly higher levels in females than in males for all tissues, except for antennae (antennae: *t* =  − 4.74, *P* = 0.01; head without antennae: *t* = 20.00, *P* = 2.73 × 10^−4^; thorax: *t* = 54.13, *P* = 4.40 × 10^−6^; abdomen: *t* = 8.65, *P* = 0.01; feet: *t* = 13.36, *P* = 1.57 × 10^−3^; wing: *t* = 88.15, *P* = 1.14 × 10^−7^).

### Expression and purification of GmolCSP11

Recombinant GmolCSP11 (rGmolCSP11) was induced with IPTG and successfully expressed in *E. coli*, mainly existing in inclusion bodies. Following denaturation, renaturation, and purification by nickel affinity chromatography, SDS-PAGE analysis showed that the purified rGmolCSP11 protein displayed a distinct single band with a molecular weight of about 13 kDa (Fig. S1).

### Fluorescent binding assays

**Figure 2:**
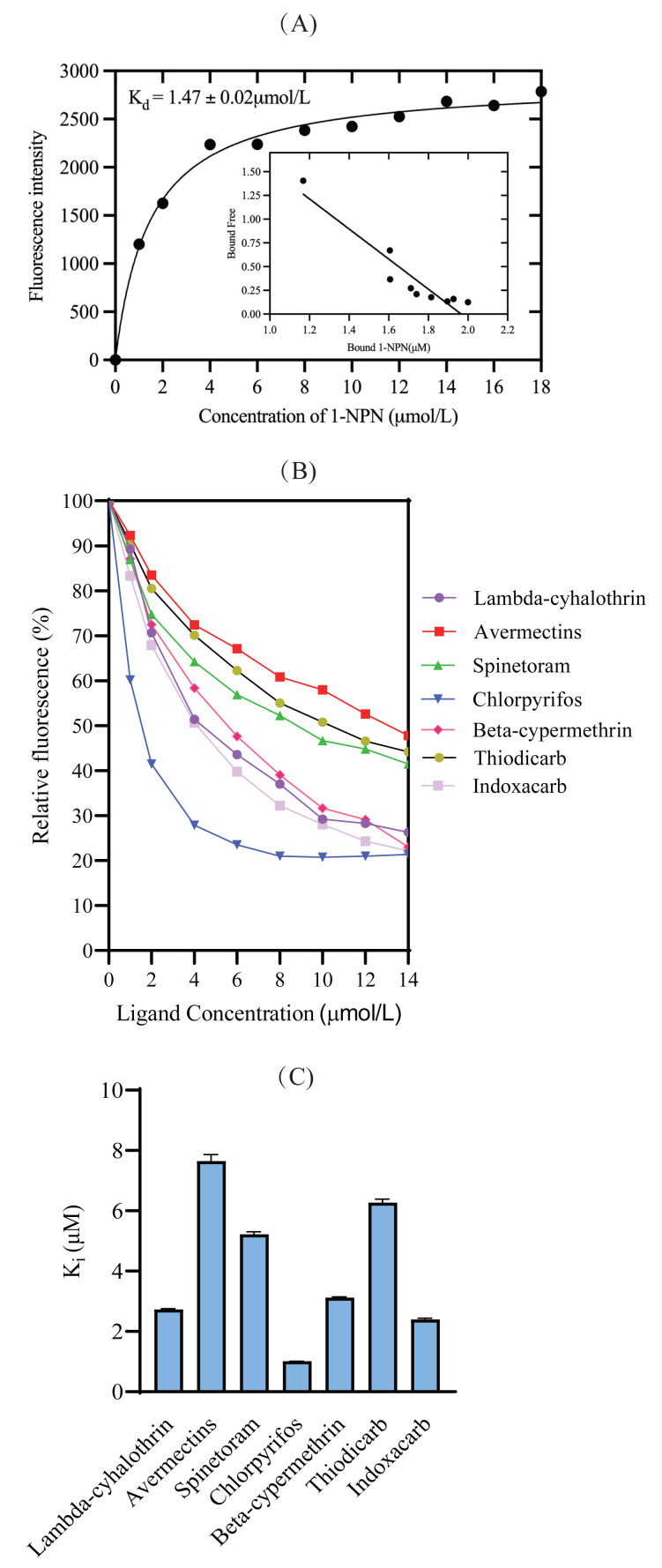
Binding characteristics of GmolCSP11 protein to different ligand. (A) The interaction of GmolCSP11 and 1-NPN. (B) Binding curves of GmolCSP11 to seven insecticide ligands. (C) Comparison of binding affinities of GmolCSP11 to seven insecticide ligands. Different lowercase and above each histogram denote significant differences in *K*_*i*_ values of GmolCSP11 binding to different insecticide ligands, respectively (one-way ANOVA combined with Tukey’s multiple comparison, *P* < 0.05). Error bars represent mean ± *SD* (*N* = 3).

The fluorescence saturation of the GmolCSP11 protein was observed with the increase of 1-NPN concentration (Fig. 2A). Therefore, the fluorescence probe 1-NPN was suitable for determining the binding properties of rGmolCSP11 with different ligands. Based on the binding data combined with the linear Scatchard equation, the dissociation constant of the rGmolCSP11/1-NPN complex was 1.47 ± 0.02 μM (Fig. 2A).

The fluorescence competitive binding assay showed that all seven insecticide ligands could successfully replace 1-NPN when the maximum final concentration of ligands was 14 μM. The initial fluorescence intensity value of the GmolCSP11/1-NPN was reduced by more than 50%, indicating that GmolCSP11 has a broad-spectrum binding spectrum for insecticides (Fig. 2B). Notably, GmolCSP11 exhibited the strongest binding affinity to chlorpyrifos (*K*_*i*_ = 1.01 ± 0.00 μM), followed by indoxacarb (*K*_*i*_ = 2.40 ± 0.02 μM), and lambda-cyhalothrin (*K*_*i*_ =2.73 ± 0.01 μM). This protein showed relatively low binding affinity to avermectin (*K*_*i*_ = 7.64  ± 0.13 μM) (Fig. 2C).

### Structural modelling and molecular docking

**Figure 3:**
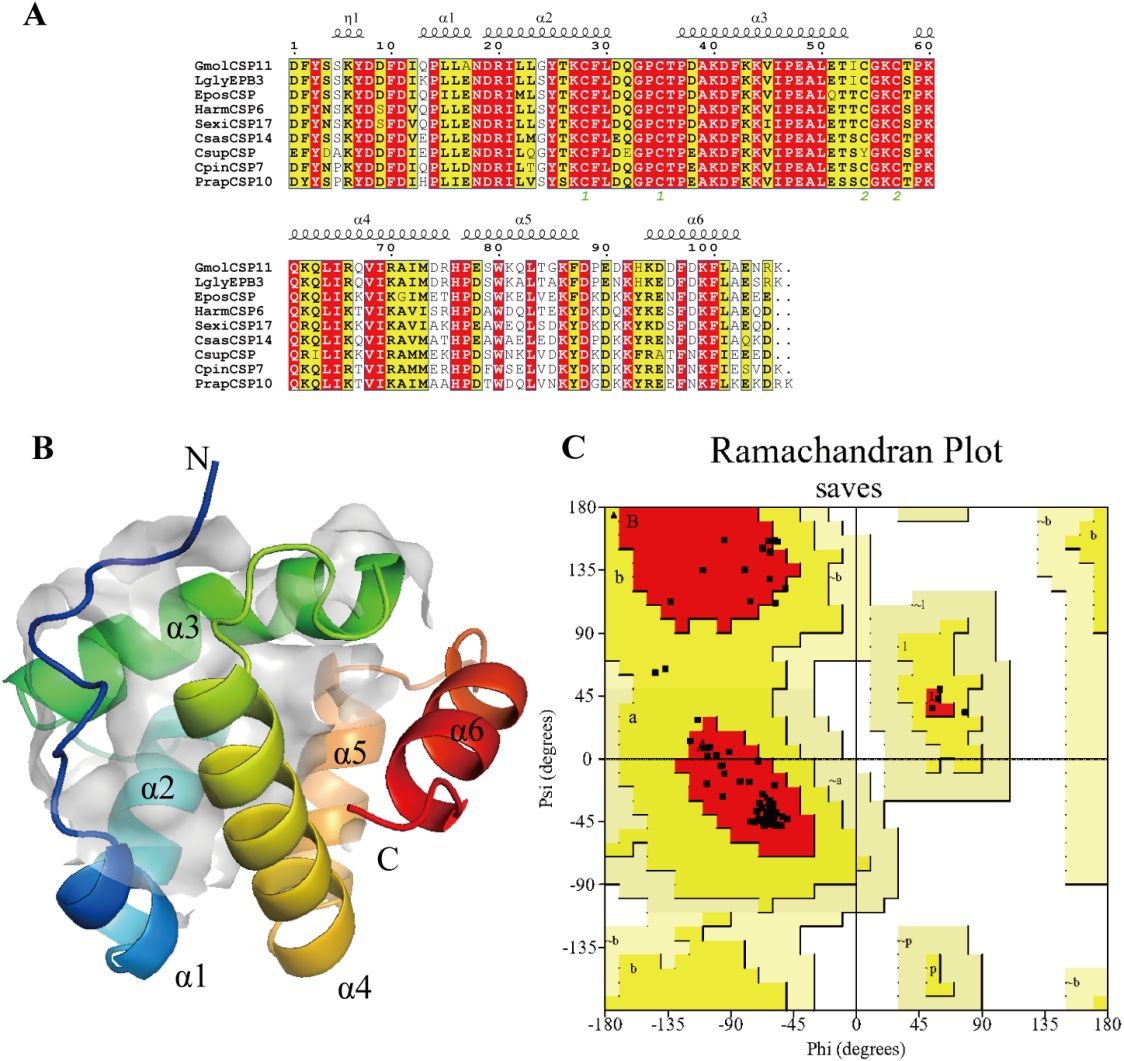
Amino acid alignment and three-dimensional structure of GmolCSP11. (A) Alignment of amino acid sequences of GmolCSP11, LgylEPB3, EposCSP, CpinCSP7, CmedCSP3, CsasCSP14, and BmorCSP11. (B) Three-dimensional structure of GmolCSP11. N-terminus, C-terminus, and helices are labelled. (C) Ramachandran plot of the GmolCSP11 model.

**Figure 4:**
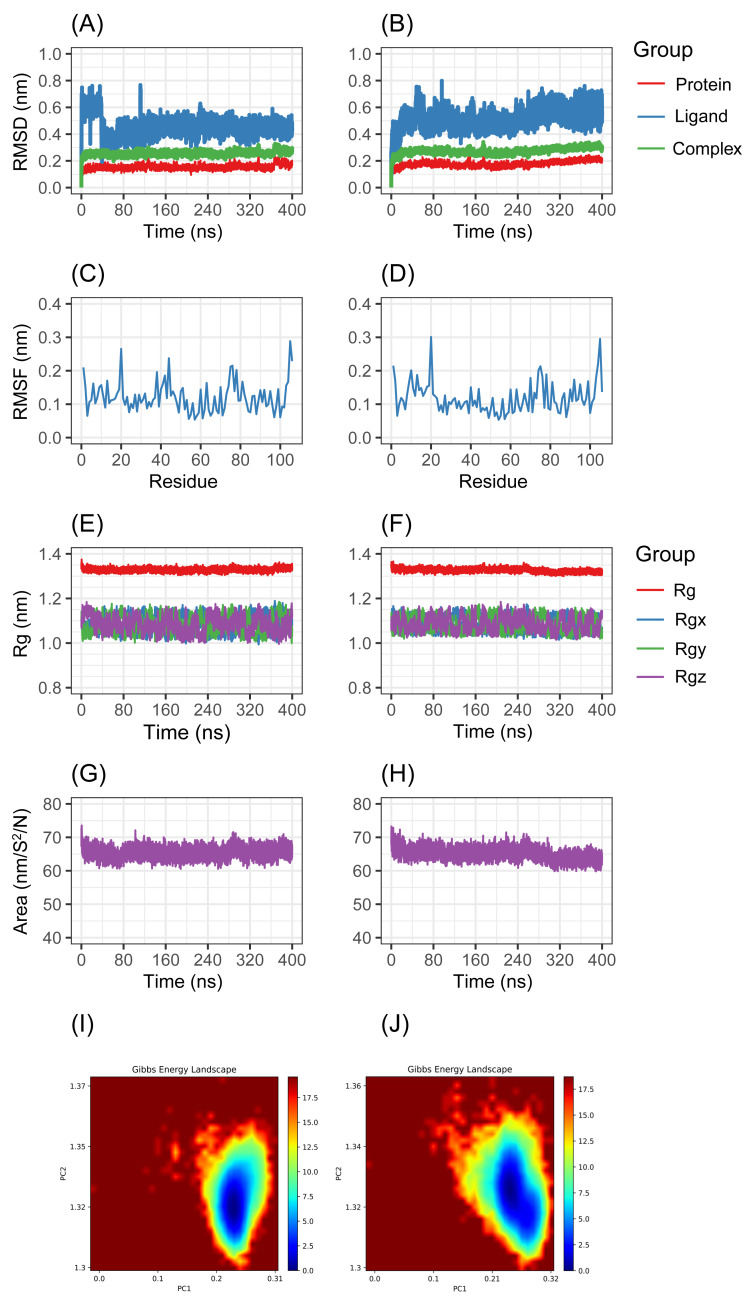
Dual-replicate molecular dynamics analysis of GmolCSP11-chlorpyrifos complex. (A–B) The Root mean square deviation (RMSD) value. (C–D) Root mean square fluctuation value (RMSF). (E–F) Radius of gyration (Rg). (G–H) Solvent accessible surface area (SASA). (I–J) Free energy landscape of the GmolCSP11–chlorpyrifos complexes as a function of RMSD (PC2) and Rg (PC1) for the GmolCSP11–chlorpyrifos during the 400 ns molecular dynamic simulations.

The 3D structure of GmolCSP11 was constructed using the alphafold DB model (A0A0K8TW01.1.A) from *Epiphyas postvittana* (Lepidoptera: Tortricidae) as the template, demonstrating 76.19% identity and 99% coverage from BLASTP search (Fig. 3A). The predicted 3D structure of GmolCSP11 features six α-helices located between residues Ile12−Glu17 (α1), Asp19−Lys30 (α2), Pro37−Thr52 (α3), Pro59−Arg75 (α4), Pro77−Phe87 (α5), and His94−Ala102 (α6), with two disulfide bonds between Cys28−Cys35 and Cys54−Cys57. Several hydrophobic residues, such as valine, alanine, leucine, proline, isoleucine, and phenylalanine, are situated within the pocket (Fig. 3B). The structural quality of GmolCSP11 was validated by PROCHECK and MolProbity. The Ramachandran plot indicated that 92.40% of the residues were allowed in the favored region, reflecting high model quality of GmolCSP11 (Fig. 4C). The analysis based on ERRAT showed a quality factor of 100, exceeding the standard quality threshold of 95 (Fig. S2).

### MD simulation of the GmolCSP11-chlorpyrifos complex

**Figure 5:**
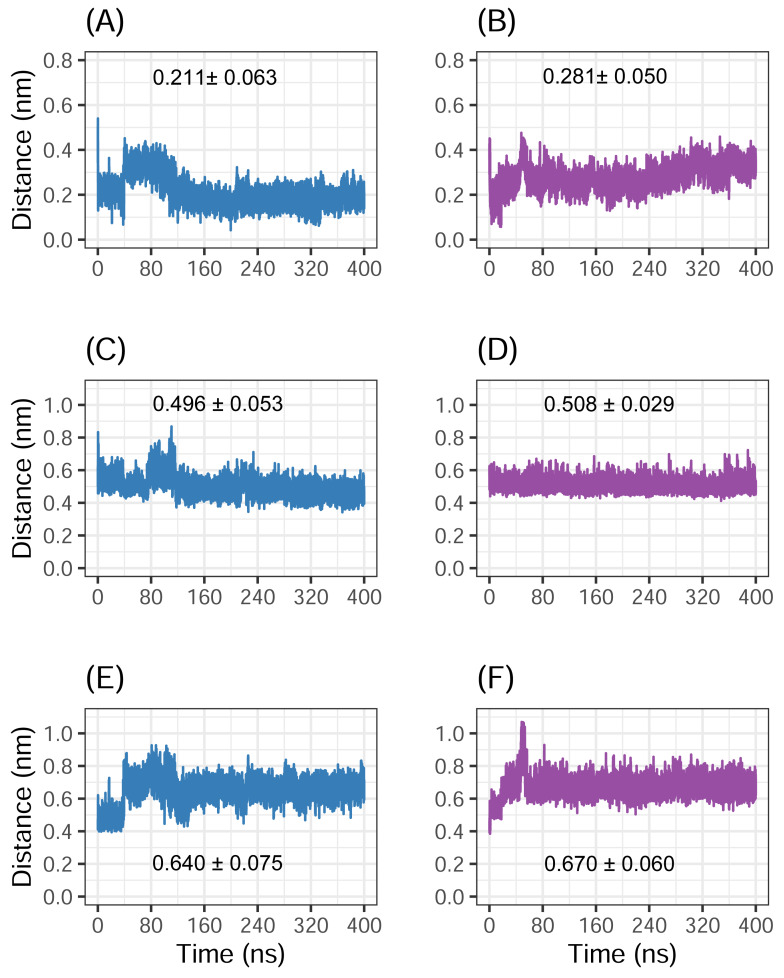
The distance fluctuation between chlorpyrifos and GmolCSP11 during two molecular dynamics simulations. (A) and (B) indicate the centroid distance between chlorpyrifos and amino acid residues within 5 Å around chlorpyrifos. (C) and (D) indicate the distance between the benzene ring of Phe42 and the pyridine ring of chlorpyrifos. (E) and (F) represent the distance between the side chain of Ile65 and the pyridine ring of chlorpyrifos. The numeric values are represented as mean ± SD.

The GmolCSP11−chlorpyrifos complex was generated by docking chlorpyrifos molecules into the binding pocket of GmolCSP11. Then, two 400 ns-MD simulations of the complex were performed, indicating that the carbon skeleton of the GmolCSP11−chlorpyrifos complex reached equilibrium at about 80 ns. The root-mean-square deviation (RMSD) was (0.255 ± 0.015) nm and (0.272 ± 0.023) nm, respectively (Figs. 4A and 4B). The modelled GmolCSP11 attained a compact structure with an average radius of gyration of (1.328 ± 0.007) nm and (1.326 ± 0.008) nm, respectively (Figs. 4E and 4F), and an average of solvent accessible surface area of (64.452 ± 1.386) nm/(S^2^ N) and (65.059 ± 1.600) nm/(S^2^ N) (Figs. 4G and 4H), indicating the stability of its 3D structure. Motion characteristics of residues in the GmolCSP11–chlorpyrifos complex were further explored based on the values of root-mean-square fluctuation (RMSF) (Figs. 4C and 4D), showing evidently higher flexibility in the N- and C-terminal loops of GmolCSP11 than in the helix region. Figs. 5I and 5J illustrated the Gibbs (FEL) free energy landscape. Additionally, by analyzing the centroid distance between chlorpyrifos and the binding pocket of GmolCSP11 (Figs. 5A and 5B), the distance between benzene ring of Phe42 and the pyridine ring of chlorpyrifos (Figs. 5C and 5D), and the distance between sidechain of Ile65 and the pyridine ring of chlorpyrifos (Figs. 5E and 5F), it was revealed that the binding mode of chlorpyrifos to GmolCSP11 remained relatively stable after 120 ns during the two simulations. The color bar represents the lowest energy (blue) to the highest (red) conformational states, suggesting that the complex gained minimum energy, aligning with the most stable conformations. These findings suggested a reliable MD trajectory for investigating the binding modes between GmolCSP11 and chlorpyrifos.

### Theoretical binding free energy calculation

Based on the trajectory sampled during a 400 ns MD simulation, the Δ*G*_*bind*_ of the GmolCSP11–chlorpyrifos complex was calculated using the MM-PBSA method. As shown in Table 1, van der Waals contributions (−179.821 ± 8.222 kJ/mol and −178.801 ± 9.035 kJ/mol, respectively) and electrostatic contributions (−19.480 ± 4.990 kJ/mol and −16.223 ± 4.813 kJ/mol, respectively) were two essential components of Δ*G*_*bind*_. The electrostatic component of the solvation free energy calculated by PB (80.988 ± 6.510 kJ/mol and 78.807 ± 6.696 kJ/mol, respectively) was unfavorable to the GmolCSP11−chlorpyrifos interaction. However, the non-polar free energy (−21.390 ± 0.476 kJ/mol and −21.387 ± 0.543 kJ/mol, respectively) calculated by the empirical model was favorable for the GmolCSP11–chlorpyrifos interaction.

**Table 1:** The calculated and experimental binding free energy for GmolCSP11-chlorpyrifos complex.

**Repeat**	**Δ*E***_*ELE*_ **(kJ/mol)**	**Δ*E***_*VDW*_ **(kJ/mol)**	**Δ*E***_*PB*_ **(kJ/mol)**	**Δ*E***_*SA*_ **(kJ/mol)**	***TdS*** **(kJ/mol)**	**Δ*G***_*bind*_ **(kJ/mol)**
1	−19.480 ± 4.990	−179.821 ± 8.222	80.988 ± 6.510	−21.390 ± 0.476	14.835	−126.578 ± 9.519
2	−16.223 ± 4.813	−178.801 ± 9.035	78.807 ± 6.696	−21.387 ± 0.543	14.614	−122.990 ± 9.647

**Notes.**

Δ*E*_*ELE*_, electrostatic energy; Δ*E*_*VDW*_, van der Waals energy; Δ*E*_*PB*_, polar solvation energy; Δ*E*_*SA*_, nonpolar solvation energy; *TdS*, temperature times entropy charge; *G*_*bind*_, Gibbs free energy.

Δ*G*_*bind*_ = Δ*E*_*ELE*_ + Δ*E*_*VDW*_ + Δ*E*_*PB*_ + Δ*E*_*SA*_ *–TdS*.

### Binding modes analysis and per-residue free energy decomposition

**Figure 6:**
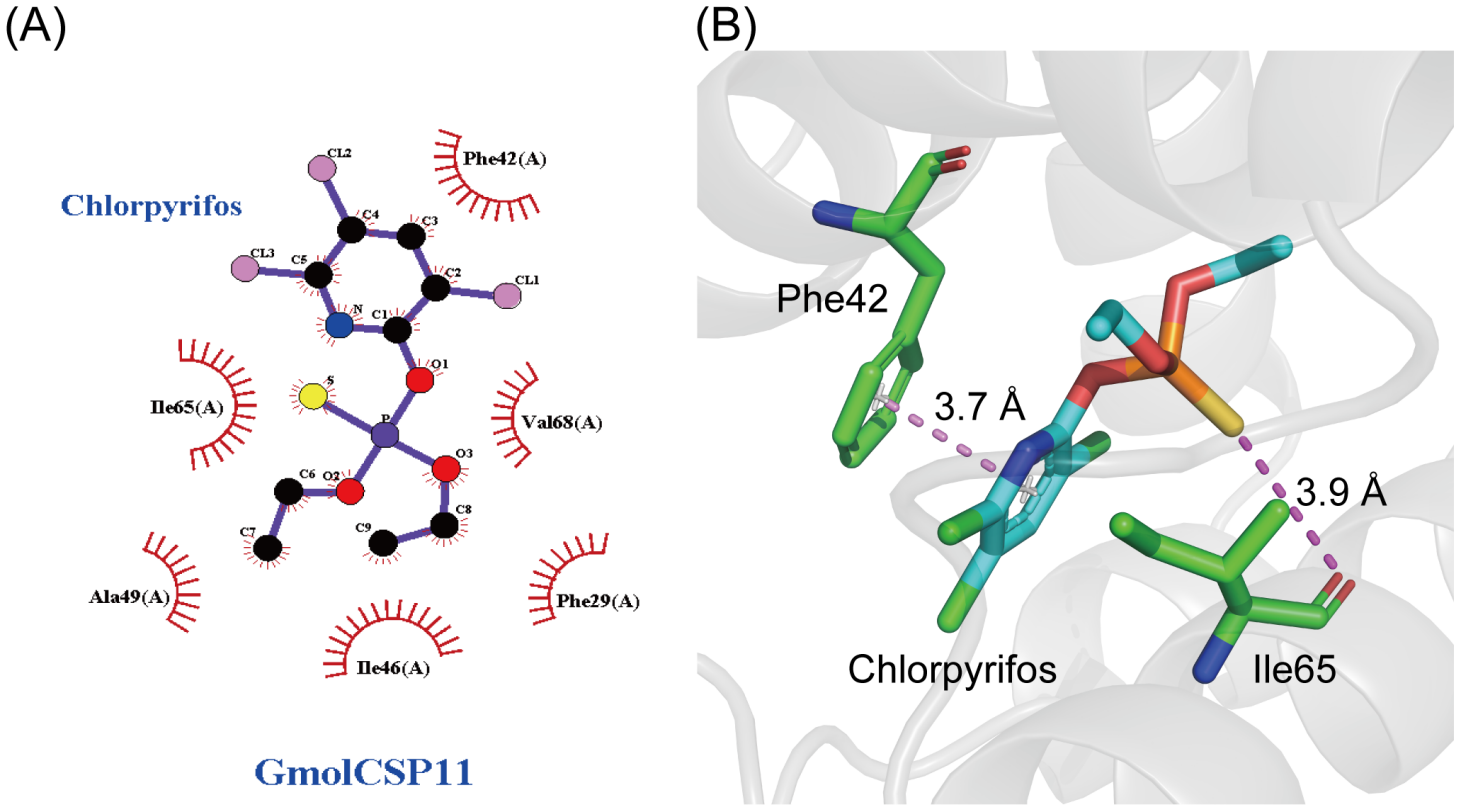
Binding mode of GmolCSP11-chlorpyrifos complex. (A) 2D-view of Key interaction and H-bond patterns at the active site observed during molecular dynamics simulations of chlorpyrifos. (B) 3D-view of Residue-ligand-interaction spectra of GmolCSP11–chlorpyrifos. Chlorpyrifos, Phe42, and Ile65 are shown in a stick model.

To analyze binding modes of the GmolCSP11–chlorpyrifos complex, we produced the representative conformation of the complex sampled during the 400 ns MD simulations. As shown in Fig. 6, a *π*-*π* conjugate interaction was detected between the benzene ring of Phe42 from GmolCSP11 and the benzene ring of chlorpyrifos, with the distance between the two benzene rings of 3.7 Å. An electrostatic interaction was detected between the sulphur atom of chlorpyrifos and the oxygen atom from the backbone of Ile65, with an S-O distance of 3.9 Å (Fig. 6).

**Figure 7:**
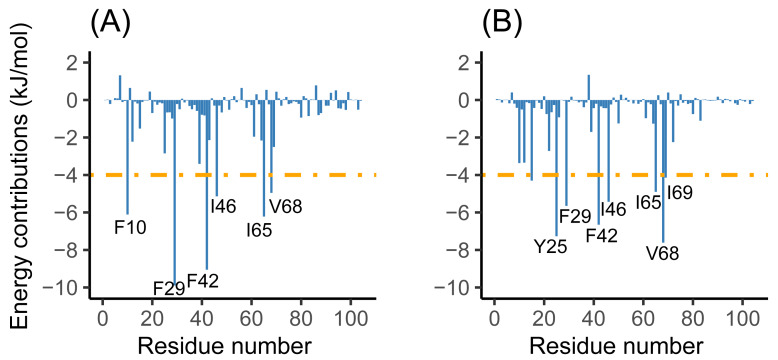
(A–B) The total energy contribution of each amino acid residue in the GmolCSP11-chlorpyrifos complex.

Five residues (Phe29, Phe42, Ile46, Ile65, Val68) contribute a prominent total free energy (*E*_*Total*_ <−4.00 kJ/mol) based on the results of two repeats of MD simulations, and the *E*_*Total*_ of Phe42 was even lower than −6.00 kJ/mol in both repeats (Fig. 7, Table S3). The *E*_*Total*_ of the above five residues dominantly derives from the van der Waals energy (*E*_*VDW*_), especially with *E*_*VDW*_ being −2.85, −9.89, −9.05, −5.14, −6.21, and −4.95 kJ/mol for Try25, Phe29, Phe42, Ile46, Ile65, and Val68, respectively, for the first MD simulations, and being −7.27, −5.65, −6.65, −5.42, −4.90, and −7.61 kJ/mol, respectively, for the second MD simulations (Table S3).

### Binding affinities of GmolCSP11 mutants

To determine the essential binding sites contributing to GmolCSP11–chlorpyrifos interaction, the four residues listed in Table 2 (Tyr25, Phe42, Leu64, and Ile65) were mutated by site-directed mutation. The affinity of GmolCSP11 for chlorpyrifos was significantly decreased when Tyr25, Phe42, Leu64, or Ile65 were altered to alanine (Table 3). The results showed that mutation of Phe42 had the most significant adverse effect on GmolCSP11 binding to chlorpyrifos, with *K*_*i*_ increasing from 1.01 to 1.89 μM (Table 2, Fig. S2), primarily attributed to the loss of a *π*-*π* conjugate interaction between the side chain of Phe42 and the benzene ring of chlorpyrifos. Furthermore, MD-simulation and per-residue free energy decomposition analysis revealed that Ile65 exhibited the lowest total free energy. Consequently, the binding affinity of GmolCSP11-I65A mutant protein for chlorpyrifos was only marginally affected.

**Table 2:** Binding capacity of the wild and mutant type recombinant GmolCSP11 protein with chlorpyrifos.

**Proteins**	***IC***_50_ **(μM)**	***K***_*i*_ **(μM)**
GmolCSP11 WT GmolCSP11 Y25A	1.35 ± 0.02 2.93 ± 0.06	1.01 ± 0.00 a 1.38 ± 0.02 bc
GmolCSP11 F42A	4.15 ± 0.01	1.89 ± 0.01 d
GmolCSP11 L64A	3.43 ± 0.12	1.38 ± 0.04 c
GmolCSP11 I65A	2.96 ± 0.04	1.28 ± 0.02 b

**Notes.**

Different lowercase letters indicate that there are significant differences of Ki (mean ± SD) among wild-type GmolCSP11 and mutated GmolCSP11 by using one-way analysis of variance (ANOVA) with Tukey’s multiple comparisons (*P* < 0.05).

**Table 3:** Binding energy of mutated GmolCSP11-c hlorpyrifos complex.

**Protein**	Y25A	F42A	L64A	I65A
**ΔΔG**_*mut*_ ** (kJ/mol)**	14.120	13.488	4.374	4.021

## Discussion

The results of RT-qPCR indicated that *GmolCSP11* was expressed at notably higher levels in antennae compared to other tissues, which was similar to the tissue expression pattern of *CsasCSP14* from the peach fruit moth, *Carposina sasakii* (Liu et al., 2021), and CmedCSP3 from *Cnaphalocrocis medinali* (Zeng et al., 2015). Additionally, moderate expression levels were observed in the heads and wings of females, consistent with the expression of *CsasCSP14* (Liu et al., 2021). A similar expression pattern between *G. molesta* and *C. sasakii* suggests a comparable function of CSPs in this gene cluster. However, when applying a homologous search using BLASTP search on NCBI, most members of this cluster were called “ejaculatory bulb-specific protein 3-like”, which limited the functional research of CSPs in this cluster.

Recent studies have demonstrated that insect CSPs are capable of binding to insecticide molecules (Lin, Mao & Zhang, 2018; Xu et al., 2022; Li et al., 2023b; Yao, Liang & Chen, 2023; Gao et al., 2023a; Yin et al., 2023; Yao et al., 2024). Certain CSPs exhibited specificity towards particular insecticides. For example, RpCSP7 from *R. padi* firmly bound *λ*-cyhalothrin, but lacks affinity to bifenthrin and deltamethrin (Gao et al., 2023a). PxCSP1 from *P. xylostella* had a strong binding affinity to indoxacarb, while weakly to *λ*-cyhalothrin, and did not show binding affinities to other tested insecticides (Li et al., 2023b). In contrast, some other CSPs had a broad binding spectrum to insecticides. For instance, RhorCSP3 from *Rhaphuma horsfieldi*, which is enriched in the antennae, binds ten different insecticides, whereas RhorCSP1 and RhorCSP2 exhibit a more limited binding spectrum (Yao et al., 2024). DcCSP4 and DcCSP8 from *Diaphorina citri* conferred dinotefuran and thiamethoxam, respectively (Li et al., 2025; Liu et al., 2025). Three CSPs from *Bradysia odoriphaga* contributed to chlorfluazuron tolerance (Ma et al., 2025). In the current study, GmolCSP11 exhibited a broad binding spectrum to insecticides, with *K*_*i*_ values for all seven tested insecticide ligands below 10 μM (Fig. 3C). These findings suggest that CSPs play a role beyond merely detecting and combining volatile chemical molecules from host/non-host plants; they also participate in insecticide resistance.

The results of fluorescent binding assays showed that chlorpyrifos acted as the most favorable ligand for GmolCSP11 among seven insecticides. Two cuticle-enriched CSPs in *Spodoptera frugiperda*, namely SfruCSP1 and SfruCSP2, displayed high binding affinities to chlorfenapyr, chlorpyrifos, and indoxacarb, with the lowest *K*_*i*_ to chlorpyrifos for both proteins (Wang et al., 2024). Recombinant SlCSP5 strongly bound in *Spodoptera litura* Fabricius. Silencing of SlCSP5 by dsRNA increased the mortality of *S. litura* larvae when exposed to chlorpyrifos (Dong et al., 2026). RpCSP1 from *R. padi* has a higher affinity for chlorpyrifos compared to the other insecticides, with a *K*_*i*_ value of 4.763 ± 0.491 μM (Gao et al., 2024a). Collectively, several CSPs are involved in the tolerance of broad-spectrum organophosphate chlorpyrifos for insects.

To investigate the potential binding mechanisms of GmolCSP11 to its favoured ligand, chlorpyrifos, we performed MD simulations. The results demonstrated that GmolCSP11 strongly bound chlorpyrifos with the binding energy of −126.578 ± 9.519 kJ/mol and −122.990 ± 9.647 kJ/mol, indicating a stable interaction between GmolCSP11 and chlorpyrifos (Table 1). Although RMSD for the ligand (0.255 ± 0.015 nm for rep. 1 and 0.272 ± 0.023 nm for rep. 2) (Figs. 5A and 5B) and the centroid distance between chlorpyrifos and amino acid residues within 5 Å  around chlorpyrifos were slightly higher in the second repeat (0.211 ± 0.063 nm for rep. 1 and 0.281 ± 0.050 nm for rep. 2) (Figs. 6A and 6B), the distance between the benzene ring of Phe42 and the pyridine ring of chlorpyrifos (0.496 ± 0.053 nm for rep. 1 and 0.508 ± 0.029 nm for rep. 2) (Figs. 6C and 6D) and the distance between the side chain of Ile65 and the pyridine ring of chlorpyrifos (0.640 ± 0.075 nm for rep. 1 and 0.670 ± 0.060 nm for rep. 2) (Figs. 6E and 6F) kept stable. In most instances, the stability of the distances between the sidechains of key residues and specific ligand atoms is more critical than the center-of-mass distances. This is because center-of-mass distances, as well as metrics such as RMSD, SASA, and hydrogen bond counts, are often dominated by contributions from the protein, which usually remains relatively rigid, thereby masking the dynamics of the more mobile ligand.

The MM/PBSA approach was employed to calculate the free energy of each residue contributing to the stability of the protein-ligand complex (Valdés-Tresanco et al., 2021). The computational simulations demonstrated that Tyr25, Phe42, Leu64, and Ile65 were crucial binding sites for GmolCSP11 in interaction with chlorpyrifos. The affinity of GmolCSP11 for chlorpyrifos was significantly decreased when Tyr25, Phe42, Leu64, or Ile65 were mutated to alanine (Table 2). Based on the alanine scanning result (Table 3), the Tyr25 and Phe42 mutation strongly destabilized the complex (ΔΔ*G*_*mut*_ > 12 kJ/mol), while Leu64 and Ile65 mutation weakly destabilized the complex (2 kJ/mol < ΔΔ*G*_*mut*_ < 8 kJ/mol). The Phe42 mutation had the greatest negative effect on GmolCSP11 binding to chlorpyrifos, largely attributed to the loss of a *π*-*π* interaction between the side chain of Phe42 and the benzene ring of chlorpyrifos. Furthermore, although MD-simulations and per-residue free-energy decomposition analysis revealed that Ile65 exhibited the lowest total free energy among these four residues, the binding affinity of GmolCSP11-I65A mutant protein for chlorpyrifos was only marginally affected. This minimal impact may be due to the synergistic hydrophobic interactions among four hydrophobic residues, Leu64, Ile65, Val68, and Ile69, which are spatially close. Mutating Ile65 alone is insufficient to significantly disrupt the GmolCSP11–chlorpyrifos complex.

## Conclusions

In this research, antennae-enriched chemosensory protein GmolCSP11 from *G. molesta* exhibited strong binding affinities to seven insecticides of diverse types, with particularly high affinity for chlorpyrifos. The results of molecular docking and MD-simulations suggested that Tyr25, Phe42, Leu64, and Ile65 were considered potential crucial amino acid residues interacting with chlorpyrifos. Among them, the site-directed mutagenesis assays highlighted Phe42 as a crucial binding site for chlorpyrifos. These findings contribute valuable insights into how moth CSPs recognize and sequester insecticides, offering potential avenues for developing novel pest management strategies.

## Supplemental Information

10.7717/peerj.21510/supp-1 Supplemental Information 1 SDS-PAGE analysis of wild-type and site-mutant GmolCSP11 (A) wild-type; (B) Y25A; (C) F42A; (D) L64A; (E) I65A. M: Protein molecular weight marker; 1. Non-induced recombinant protein; 2. Induced recombinant protein; 3. recombinant protein supernatant; 4. recombinant protein pellet; 5. Purified recombinant protein.

10.7717/peerj.21510/supp-2 Supplemental Information 2 ERRAT plot for GmolCSP11

10.7717/peerj.21510/supp-3 Supplemental Information 3 Binding affinities of the GmolCSP11 mutants to Chlorpyrifos

10.7717/peerj.21510/supp-4 Supplemental Information 4 The primer sequences of GmolCSP11 for RT-qPCR and site-directed mutagenesis

10.7717/peerj.21510/supp-5 Supplemental Information 5 The sources of seven insecticide ligands used in fluorescence binding assays

10.7717/peerj.21510/supp-6 Supplemental Information 6 Energy contribution of residues within 5 Å around chlorpyrifos in the GmolCSP11−chlorpyrifos complex

10.7717/peerj.21510/supp-7 Supplemental Information 7 Raw data

10.7717/peerj.21510/supp-8 Supplemental Information 8 MIQE checklist

## Additional Information and Declarations

### Data Availability

The following information was supplied regarding data availability: The raw measurements are available in the Supplemental Files. The raw sequence data are available at GenBank: KR003783.1, ALC79597.1

## References

[ref-1] Abraham MJ, Murtola T, Schulz R, Páll S, Smith JC, Hess B, Lindahl E (2015). GROMACS: high performance molecular simulations through multi-level parallelism from laptops to supercomputers. SoftwareX.

[ref-2] Balabanidou V, Grigoraki L, Vontas J (2018). Insect cuticle: a critical determinant of insecticide resistance. Current Opinion in Insect Science.

[ref-3] Casida JE, Durkin KA (2013). Neuroactive insecticides: targets, selectivity, resistance, and secondary effects. Annual Review of Entomology.

[ref-4] Cui Z, Liu Y, Wang G, Zhou Q (2022). Identification and functional analysis of a chemosensory protein from *Bactrocera minax* (Diptera: Tephritidae). Pest Management Science.

[ref-5] Delgado J, Radusky LG, Cianferoni D, Serrano L (2019). FoldX 5.0: working with RNA, small molecules and a new graphical interface. Bioinformatics.

[ref-6] Dong X, Tan T, Pei Y, Xiang F, Li C (2026). An inducible chemosensory protein (SlCSP5) in the integument is associated with chlorpyrifos tolerance in *Spodoptera litura* Fabricius (Lepidoptera: Noctuidae). Pesticide Biochemistry and Physiology.

[ref-7] Duan SG, Lv CL, Liu JH, Yi SC, Yang RN, Liu A, Wang MQ (2022). NlugOBP8 in *Nilaparvata lugens* involved in the perception of two terpenoid compounds from rice plant. Journal of Agricultural and Food Chemistry.

[ref-8] Duarte F, Calvo MV, Borges A, Scatoni IB (2015). Geostatistics and geographic information systems to study the spatial distribution of *Grapholita molesta* (Busck) (Lepidoptera: Tortricidae) in peach fields. Neotropical Entomology.

[ref-9] Ebrahim SAM, Dweck HKM, Stökl J, Hofferberth JE, Trona F, Weniger K, Rybak J, Seki Y, Stensmyr MC, Sachse S, Hansson BS, Knaden M (2015). *Drosophila* avoids parasitoids by sensing their semiochemicals *via* a dedicated olfactory circuit. PLOS Biology.

[ref-10] Edgar RC (2004). MUSCLE: multiple sequence alignment with high accuracy and high throughput. Nucleic Acids Research.

[ref-11] Gao P, Tan JJ, Peng X, Qu MJ, Chen M (2024a). Key residues involved in the interaction between chlorpyrifos and a chemosensory protein in *Rhopalosiphum padi*: implication for tracking chemical residues *via* insect olfactory proteins. Science of the Total Environment.

[ref-12] Gao P, Tan JJ, Peng X, Song Y, Qu MJ, Chen MH (2024b). Expression pattern of *RpCSP6* from *Rhopalosiphum padi* and its binding mechanism with deltamethrin: insights into chemosensory protein-mediated insecticide resistance. Journal of Agricultural and Food Chemistry.

[ref-13] Gao P, Tan JJ, Su S, Wang SJ, Peng X, Chen MH (2023a). Overexpression of the chemosensory protein *CSP7* gene contributed to lambda-cyhalothrin resistance in the bird cherry-oat aphid *Rhopalosiphum padi*. Journal of Agricultural and Food Chemistry.

[ref-14] Gao P, Zhang S, Tan JJ, Li X, Chen MH (2023b). Chemosensory proteins are associated with thiamethoxam tolerance in bird cherry-oat aphid *Rhopalosiphum padi*. Pesticide Biochemistry and Physiology.

[ref-15] Gouet P, Robert X, Courcelle E (2003). ESPript/ENDscript: extracting and rendering sequence and 3D information from atomic structures of proteins. Nucleic Acids Research.

[ref-16] Guo W, Wang X, Ma Z, Xue L, Han J, Yu D, Kang L (2011). *CSP* and *takeout* genes modulate the switch between attraction and repulsion during behavioral phase change in the migratory locust. PLOS Genetics.

[ref-17] Han H, Yang Y, Hu J, Wang Y, Zhao Z, Ma R, Gao L, Guo Y (2022). Identification and characterization of CYP6 family genes from the oriental fruit moth (*Grapholita molesta*) and their responses to insecticides. Insects.

[ref-18] He H, Crabbe MJC, Ren Z (2023). Genome-wide identification and characterization of the chemosensory relative protein genes in Rhus gall aphid *Schlechtendalia chinensis*. BMC Genomics.

[ref-19] Jing D, Zhang T, Bai S, He K, Prabu S, Luan J, Wang Z (2020). Sexual-biased gene expression of olfactory-related genes in the antennae of *Conogethes pinicolalis* (Lepidoptera: Crambidae). BMC Genomics.

[ref-20] Kanga LH, Pree DJ, Van Lier JL, Walker GM (2003). Management of insecticide resistance in oriental fruit moth (*Grapholita molesta*; Lepidoptera: Tortricidae) populations from Ontario. Pest Management Science.

[ref-21] Kim S, Chen J, Cheng T, Gindulyte A, He J, He S, Li Q, Shoemaker BA, Thiessen PA, Yu B, Zaslavsky L, Zhang J, Bolton EE (2023). PubChem 2023 update. Nucleic Acids Research.

[ref-22] Lartigue A, Campanacci V, Roussel A, Larsson AM, Jones TA, Tegoni M, Cambillau C (2002). X-ray structure and ligand binding study of a moth chemosensory protein. Journal of Biological Chemistry.

[ref-23] Leal WS (2013). Odorant reception in insects: roles of receptors, binding proteins, and degrading enzymes. Annual Review of Entomology.

[ref-24] Li G, Du J, Li Y, Wu J (2015). Identification of putative olfactory genes from the oriental fruit moth *Grapholita molesta via* an antennal transcriptome analysis. PLOS ONE.

[ref-25] Li Q, Hu WW, Fang A, Wang ZB, Yuan XF, Sun Y, Zou ZH, Chen N, Jing TX, Liu YX, Chen W, Yu HZ, Lu ZJ, Liu XQ (2025). Chemosensory protein 8 confers thiamethoxam resistance in *Diaphorina citri*. Pesticide Biochemistry and Physiology.

[ref-26] Li J, Jia Y, Zhang D, Li Z, Zhang S, Liu X (2023a). Molecular identification of carboxylesterase genes and their potential roles in the insecticides susceptibility of *Grapholita molesta*. Insect Molecular Biology.

[ref-27] Li Y, Ni S, Wang Y, Li R, Sun H, Ye X, Tian Z, Zhang Y, Liu J (2023b). The chemosensory protein 1 contributes to indoxacarb resistance in *Plutella xylostella* (L.). Pest Management Science.

[ref-28] Li F, Venthur H, Wang S, Homem RA, Zhou JJ (2021). Evidence for the involvement of the chemosensory protein *AgosCSP5* in resistance to insecticides in the cotton aphid, *Aphis gossypii*. Insects.

[ref-29] Lin X, Mao Y, Zhang L (2018). Binding properties of four antennae-expressed chemosensory proteins (CSPs) with insecticides indicates the adaption of Spodoptera litura to environment. Pesticide Biochemistry and Physiology.

[ref-30] Liu C, Chen Y, Xie X, Hu H, Wu J, Ye C, Tang R, Wu Z, Shu B (2025). The chemosensory protein CSP4 increased inotefuran tolerance in *Diaphorina citri* adults. Pesticide Biochemistry and Physiology.

[ref-31] Liu G, Ma H, Xie H, Xuan N, Guo X, Fan Z, Rajashekar B, Arnaud P, Offmann B, Picimbon JF (2016). Biotype characterization, developmental profiling, insecticide response and binding property of *Bemisia tabaci* chemosensory proteins: role of CSP in insect defense. PLOS ONE.

[ref-32] Liu X, Sun L, Tong Z, Zhang H, Yan W, Yue Q, Qiu G (2021). Cloning and molecular docking of the *Carposina sasakii* chemosensory protein gene, CsasCSP14. Chinese Journal of Applied Entomology.

[ref-33] Liu Y, Yang X, Gan J, Chen S, Xiao ZX, Cao Y (2022). CB-Dock2: improved protein–ligand blind docking by integrating cavity detection, docking and homologous template fitting. Nucleic Acids Research.

[ref-34] Ma X, Zeng J, Dai W, Zhang C (2025). Three chemosensory proteins contribute to chlorfluazuron tolerance in *Bradysia odoriphaga*. Journal of Agricultural and Food Chemistry.

[ref-35] Maleszka J, Forêt S, Saint R, Maleszka R (2007). RNAi-induced phenotypes suggest a novel role for a chemosensory protein CSP5 in the development of embryonic integument in the honeybee (*Apis mellifera*). Development Genes and Evolution.

[ref-36] McKenna MP, Hekmat-Scafe DS, Gaines P, Carlson JR (1994). Putative *Drosophila* pheromone-binding proteins expressed in a subregion of the olfactory system. Journal of Biological Chemistry.

[ref-37] Myers CT, Hull LA, Krawczyk G (2006). Seasonal and cultivar-associated variation in oviposition preference of oriental fruit moth (Lepidoptera: Tortricidae) adults and feeding behavior of neonate larvae in apples. Journal of Economic Entomology.

[ref-38] Najar-Rodriguez A, Bellutti N, Dorn S (2013). Larval performance of the oriental fruit moth across fruits from primary and secondary hosts. Physiological Entomology.

[ref-39] Pavlidi N, Vontas J, Van Leeuwen T (2018). The role of glutathione S-transferases (GSTs) in insecticide resistance in crop pests and disease vectors. Current Opinion in Insect Science.

[ref-40] Pelosi P, Iovinella I, Felicioli A, Dani FR (2014). Soluble proteins of chemical communication: an overview across arthropods. Frontiers in Physiology.

[ref-41] Pelosi P, Iovinella I, Zhu J, Wang G, Dani FR (2018). Beyond chemoreception: diverse tasks of soluble olfactory proteins in insects. Biological Reviews.

[ref-42] Peng X, Qu MJ, Wang SJ, Huang YX, Chen C, Chen MH (2021). Chemosensory proteins participate in insecticide susceptibility in *Rhopalosiphum padi*, a serious pest on wheat crops. Insect Molecular Biology.

[ref-43] Renou M, Anton S (2020). Insect olfactory communication in a complex and changing world. Current Opinion in Insect Science.

[ref-44] Robertson HM, Martos R, Sears CR, Todres EZ, Walden KKO, Nardi JB (1999). Diversity of odourant binding proteins revealed by an expressed sequence tag project on male *Manduca sexta* moth antennae. Insect Molecular Biology.

[ref-45] Schmittgen TD, Livak KJ (2008). Analyzing real-time PCR data by the comparative CT method. Nature Protocols.

[ref-46] Showalter SA, Brüschweiler R (2007). Validation of molecular dynamics simulations of biomolecules using NMR spin relaxation as benchmarks: application to the AMBER99SB force field. Journal of Chemical Theory and Computation.

[ref-47] Teufel F, Almagro Armenteros JJ, Johansen AR, Gíslason MH, Pihl SI, Tsirigos KD, Winther O, Brunak S, Von Heijne G, Nielsen H (2022). SignalP 6.0 predicts all five types of signal peptides using protein language models. Nature Biotechnology.

[ref-48] Valdés-Tresanco MS, Valdés-Tresanco ME, Valiente PA, Moreno E (2021). gmx_MMPBSA: a new tool to perform end-state free energy calculations with GROMACS. Journal of Chemical Theory and Computation.

[ref-49] Walsh TK, Heckel DG, Wu Y, Downes S, Gordon KHJ, Oakeshott JG (2022). Determinants of insecticide resistance evolution: comparative analysis among Heliothines. Annual Review of Entomology.

[ref-50] Wang H, Zhao R, Gao J, Xiao X, Yin X, Hu S, Zhang Y, Liang P, Gu S (2024). Two cuticle-enriched chemosensory proteins confer multi-insecticide resistance in *Spodoptera frugiperda*. International Journal of Biological Macromolecules.

[ref-51] Waterhouse A, Bertoni M, Bienert S, Studer G, Tauriello G, Gumienny R, Heer FT, De Beer TAP, Rempfer C, Bordoli L, Lepore R, Schwede T (2018). SWISS-MODEL: homology modelling of protein structures and complexes. Nucleic Acids Research.

[ref-52] Wei SJ, Cao LJ, Gong YJ, Shi BC, Wang S, Zhang F, Guo XJ, Wang YM, Chen XX (2015). Population genetic structure and approximate Bayesian computation analyses reveal the southern origin and northward dispersal of the oriental fruit moth *Grapholita molesta* (Lepidoptera: Tortricidae) in its native range. Molecular Ecology.

[ref-53] Wickham H (2016).

[ref-54] Xu H, Pan Y, Li J, Yang F, Chen X, Gao X, Wen S, Shang Q (2022). Chemosensory proteins confer adaptation to the ryanoid anthranilic diamide insecticide cyantraniliprole in *Aphis gossypii* glover. Pesticide Biochemistry and Physiology.

[ref-55] Yang F, Cao LJ, Nguyen P, Ma ZZ, Chen JC, Song W, Wei SJ (2025). Hierarchical architecture of neo-sex chromosomes and accelerated adaptive evolution in tortricid moths. Genome Research.

[ref-56] Yao Q, Liang Z, Chen B (2023). Evidence for the participation of chemosensory proteins in response to insecticide challenge in *Conopomorpha sinensis*. Journal of Agricultural and Food Chemistry.

[ref-57] Yao YJ, Yin NN, Pu LM, Yang AJ, Liu NY (2024). Three chemosensory proteins enriched in antennae and tarsi of *Rhaphuma horsfieldi* differentially contribute to the binding of insecticides. Pesticide Biochemistry and Physiology.

[ref-58] Ye J, Coulouris G, Zaretskaya I, Cutcutache I, Rozen S, Madden TL (2012). Primer-BLAST: a tool to design target-specific primers for polymerase chain reaction. BMC Bioinformatics.

[ref-59] Yi X, Qi J, Zhou X, Hu MY, Zhong GH (2017). Differential expression of chemosensory-protein genes in midguts in response to diet of *Spodoptera litura*. Scientific Reports.

[ref-60] Yin NN, Yao YJ, Liang YL, Wang ZQ, Li YH, Liu NY (2023). Functional characterization of four antenna-biased chemosensory proteins in *Dioryctria abietella* reveals a broadly tuned olfactory DabiCSP1 and its key residues in ligand-binding. Pesticide Biochemistry and Physiology.

[ref-61] Zeng FF, Zhao ZF, Yan MJ, Zhou W, Zhang Z, Zhang A, Lu ZX, Wang MQ (2015). Identification and comparative expression profiles of chemoreception genes revealed from major chemoreception organs of the rice leaf folder, *Cnaphalocrocis medinalis* (Lepidoptera: Pyralidae). PLOS ONE.

[ref-62] Zhang X, Wang X, Guo Z, Liu X, Wang P, Yuan X, Li Y (2022). Antibiotic treatment reduced the gut microbiota diversity, prolonged the larval development period and lessened adult fecundity of *Grapholita molesta* (Lepidoptera: Tortricidae). Insects.

[ref-63] Zhang S, Zhang D, Jia Y, Li J, Li Z, Liu X (2023). Molecular identification of glutathione S-transferase genes and their potential roles in insecticides susceptibility of *Grapholita molesta*. Journal of Applied Entomology.

[ref-64] Zheng R, Xie M, Keyhani NO, Xia Y (2023). An insect chemosensory protein facilitates locust avoidance to fungal pathogens *via* recognition of fungal volatiles. International Journal of Biological Macromolecules.

[ref-65] Zhou Z, Luo Y, Wang X, He J, Zhou Q (2024). Identification and sex expression profiles of candidate chemosensory genes from *Atherigona orientalis via* the antennae and leg transcriptome analysis. Comparative Biochemistry and Physiology Part D: Genomics and Proteomics.

