# Supplementary figures and images for: Binding characteristics of chemosensory protein 11 from Grapholita molesta Busck (Lepidoptera: Tortricidae) to insecticides
Source: PeerJ. 2026 Jul 20;14:e21510. doi: 10.7717/peerj.21510 (PMC13394210; doi:10.7717/peerj.21510)

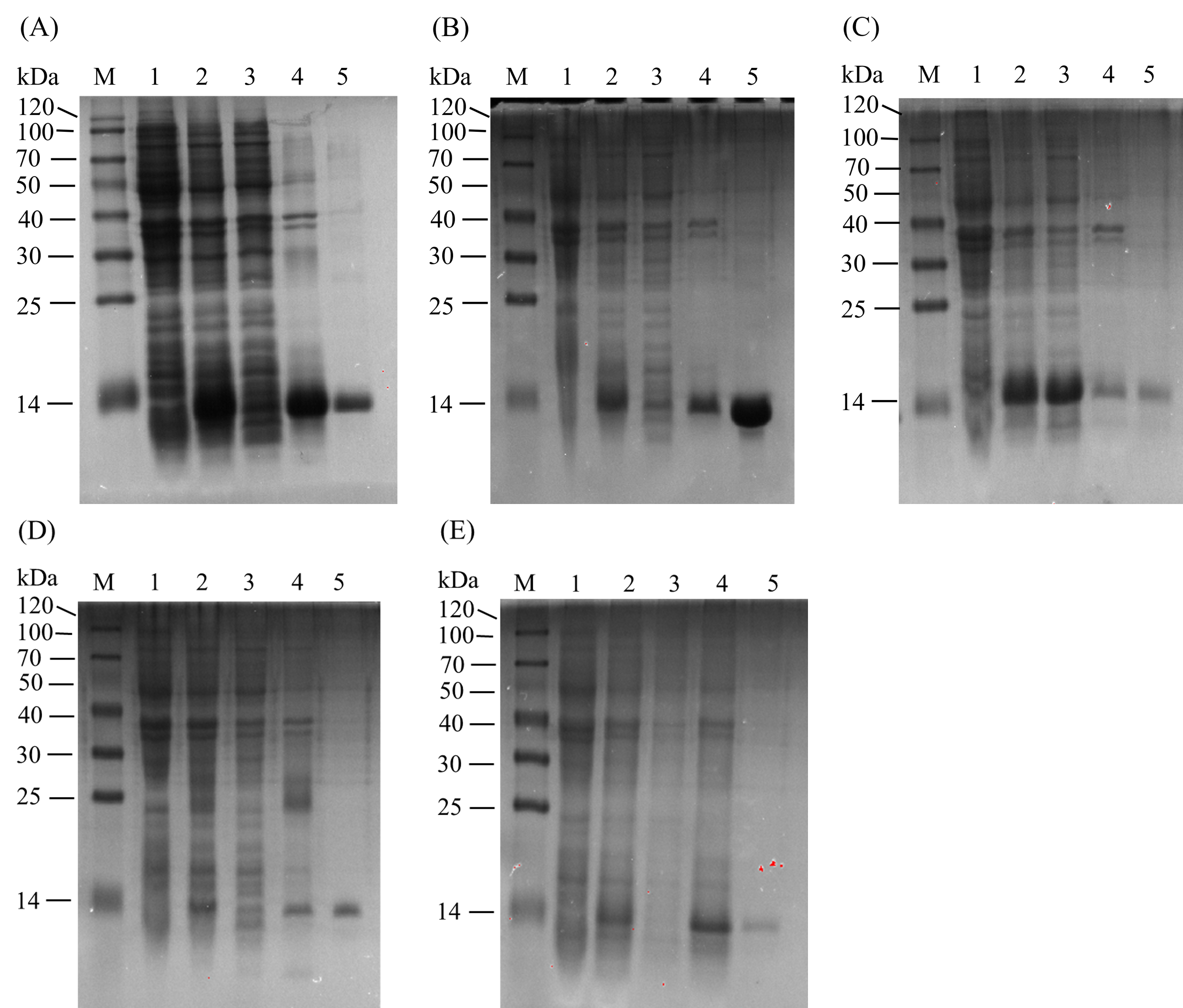

Supplement: Supplemental Information 1 — (A) wild-type; (B) Y25A; (C) F42A; (D) L64A; (E) I65A. M: Protein molecular weight marker; 1. Non-induced recombinant protein; 2. Induced recombinant protein; 3. recombinant protein supernatant; 4. recombinant protein pellet; 5. Purified recombinant protein. [file peerj-14-21510-s001.png]

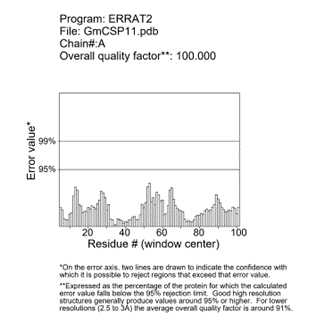

Supplement: Supplemental Information 2 [file peerj-14-21510-s002.png]

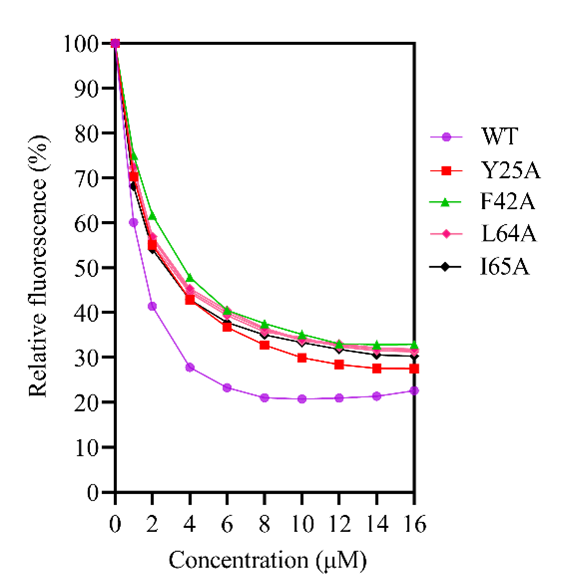

Supplement: Supplemental Information 3 [file peerj-14-21510-s003.png]
