# Supplementary material for: Binding characteristics of chemosensory protein 11 from Grapholita molesta Busck (Lepidoptera: Tortricidae) to insecticides
Source: PeerJ. 2026 Jul 20;14:e21510. doi: 10.7717/peerj.21510 (PMC13394210; doi:10.7717/peerj.21510)
Supplement: Supplemental Information 4 [file peerj-14-21510-s004.docx]

**Table S1 The primer sequences of GmolCSP11 for RT-qPCR and site-directed mutagenesis**

| **Primer Name** | **Primer Sequence (5’-3’)** |
| --- | --- |
| For RT-qPCR |  |
| qGmolCSP11-sense | GTCACCGCCGACTTCTAC |
| qGmolCSP11-antisense | GATCAGCTGCTTTTGTTT |
| β-actin-Sense | CTTTCACCACCACCGCTG |
| β-actin-Anti-sense | CGCAAGATTCCATACCCA |
| For site-directed mutagenesis |  |
| Y25→A25 TAC→GCC |  |
| Y25A-Sense | GGCGCCACCAAGTGCTTCCTCGACCAGGGGCCGTG |
| Y25A-Antisense | GGAAGCACTTGGTGGCGCCCAGCAGGATCCTGTCG |
| F42→A42 TTT→GCT |  |
| Y25A-Sense | CTAAGGACGCTAAAAAAGTGATCCCAGAAGCCCTAG |
| Y25A-Antisense | CTTTTTTAGCGTCCTTAGCGTCGGGAGTGCACGGCC |
| L64→A64 CTG→GCG |  |
| L64A-Sense | CAAAAGCAGGCGATCAGGCAAGCCATTAGAGCCATC |
| L64A-Antisense | CTGATCGCCTGCTTTTGTTTAGGCGTGCATTTCCCG |
| I65→A65 ATC→GCC |  |
| L64A-Sense | CAGCTGGCCAGGCAAGCCATTAGAGCCATCATG |
| L64A-Antisense | CTTGCCTGGCCAGCTGCTTTTGTTTAGGCGTGC |
