# Supplementary material for: Binding characteristics of chemosensory protein 11 from Grapholita molesta Busck (Lepidoptera: Tortricidae) to insecticides
Source: PeerJ. 2026 Jul 20;14:e21510. doi: 10.7717/peerj.21510 (PMC13394210; doi:10.7717/peerj.21510)
Supplement: Supplemental Information 5 [file peerj-14-21510-s005.docx]

**Table S2 The sources of seven insecticide ligands used in fluorescence binding assays.**

| **Chemical compounds** | **Molecular weight** | **Formula** | **Purity** | **Source** |
| --- | --- | --- | --- | --- |
| Chlorpyrifos | 350.59 | C_9_H_11_Cl_3_NO_3_PS | 97.0% (AR) | Sigma-Aldrich |
| Beta-cypermethrin | 416.30 | C_22_H_19_Cl_2_NO_3_ | 95.9% (AR) | Shanghai Yudiao Chemistry Technology Co.,Ltd |
| Lambda-cyhalothrin | 449.85 | C_23_H_19_ClF_3_NO_3_ | 96.0% (AR) | Jiangsu Ruidong Pesticide Co., Ltd. |
| Thiodicarb | 354.47 | C_10_H_18_N_4_O_4_S_3_ | 96.0% (AR) | aladdin |
| Abamectin | 887.11 | C_49_H_74_O_14_ | 98.0% (AR) | TopScience |
| Spinetoram | 748.00 | C_42_H_69_NO_10_ | >98.0% (AR) | MedBio |
| Indoxacarb | 527.83 | C_22_H_17_ClF_3_N_3_O_7_ | 95.0% (AR) | BOC Sciences |
