# Supplementary material for: Binding characteristics of chemosensory protein 11 from Grapholita molesta Busck (Lepidoptera: Tortricidae) to insecticides
Source: PeerJ. 2026 Jul 20;14:e21510. doi: 10.7717/peerj.21510 (PMC13394210; doi:10.7717/peerj.21510)
Supplement: Supplemental Information 6 [file peerj-14-21510-s006.docx]

**Table S3** **Energy contribution of residues within 5Å around chlorpyrifos in the GmolCSP11−chlorpyrifos complex**

| **Repeat** | **Residue** | *Δ****E_ELE_* (kJ/mol)** | *Δ****E_VDW_* (kJ/mol)** | *Δ****E_PB_***  **(kJ/mol)** | *Δ****E_SA_***  **(kJ/mol)** | *Δ****E_total_***  **(kJ/mol)** |
| --- | --- | --- | --- | --- | --- | --- |
| 1 | Try7 | -0.972 | -5.372 | 8.160 | -0.501 | 1.315 |
|  | Phe10 | -0.724 | -7.518 | 2.768 | -0.631 | -6.105 |
|  | Ile12 | 0.083 | -2.230 | 0.014 | -0.088 | -2.221 |
|  | Leu15 | -0.463 | -1.311 | 0.283 | -0.033 | -1.524 |
|  | Tyr25 | -0.646 | -2.717 | 0.611 | -0.092 | -2.845 |
|  | Phe29 | -2.069 | -11.427 | 4.64 | -1.034 | -9.889 |
|  | Phe42 | -0.785 | -10.24 | 2.832 | -0.859 | -9.052 |
|  | Val45 | -0.031 | -0.4 | 0.134 | 0 | -0.297 |
|  | Ile46 | 0.016 | -4.888 | 0.254 | -0.518 | -5.136 |
|  | Ala49 | 0.091 | -0.131 | 0.2 | 0 | 0.161 |
|  | Gln61 | -0.087 | -1.302 | -0.538 | -0.028 | -1.955 |
|  | Leu64 | 1.04 | -3.952 | 0.952 | -0.195 | -2.156 |
|  | Ile65 | 0.203 | -6.232 | 0.32 | -0.511 | -6.219 |
|  | Val68 | -0.104 | -4.889 | 0.488 | -0.45 | -4.954 |
|  | Ile69 | 0.274 | -2.563 | 0.059 | -0.274 | -2.505 |
| 2 | Try7 | -0.478 | -0.854 | 1.76 | -0.025 | 0.403 |
|  | Phe10 | -0.609 | -4.461 | 2.031 | -0.325 | -3.364 |
|  | Ile12 | -0.122 | -3.170 | 0.175 | -0.223 | -3.340 |
|  | Leu15 | -0.279 | -4.06 | 0.357 | -0.318 | -4.301 |
|  | Tyr25 | 0.722 | -8.899 | 1.492 | -0.581 | -7.267 |
|  | Phe29 | -0.261 | -7.111 | 2.468 | -0.743 | -5.647 |
|  | Phe42 | -0.929 | -8.084 | 2.999 | -0.636 | -6.651 |
|  | Val45 | -0.379 | -0.263 | 0.208 | 0 | -0.433 |
|  | Ile46 | -0.793 | -4.556 | 0.366 | -0.446 | -5.429 |
|  | Ala49 | -0.234 | -0.158 | 0.294 | 0 | -0.099 |
|  | Gln61 | -0.044 | -0.421 | -0.501 | -0.006 | -0.971 |
|  | Leu64 | 0.614 | -2.697 | 0.964 | -0.139 | -1.258 |
|  | Ile65 | -0.067 | -4.82 | 0.229 | -0.241 | -4.899 |
|  | Val68 | -0.906 | -8.757 | 3.046 | -0.995 | -7.611 |
|  | Ile69 | -0.22 | -4.195 | 0.631 | -0.356 | -4.139 |

*ΔE_ELE_*, electrostatic energy; *ΔE_VDW_*, van der Waals energy; *ΔE_PB_*, polar solvation energy; *ΔE_SA_*, nonpolar solvation energy; *E_total_*, algebraic sum of four energy contributions above.
